# Professionals’ Digital Training for Child Maltreatment Prevention in the COVID-19 Era: A Pan-European Model

**DOI:** 10.3390/ijerph19020885

**Published:** 2022-01-13

**Authors:** Cristina Crocamo, Bianca Bachi, Riccardo M. Cioni, Henrike Schecke, Irja Nieminen, Lidia Zabłocka-Żytka, Małgorzata Woźniak-Prus, Francesco Bartoli, Ilaria Riboldi, Jane V. Appleton, Sarah Bekaert, Giedre Zlatkute, Emmanuelle Jouet, Giovanni Viganò, Michael Specka, Norbert Scherbaum, Eija Paavilainen, Alexander Baldacchino, Giuseppe Carrà

**Affiliations:** 1 Department of Medicine and Surgery, University of Milano-Bicocca, Via Cadore 48, 20900 Monza, Italy; b.bachi@campus.unimib.it (B.B.); r.cioni1@campus.unimib.it (R.M.C.); francesco.bartoli@unimib.it (F.B.); i.riboldi1@campus.unimib.it (I.R.); giuseppe.carra@unimib.it (G.C.); 2 LVR-Hospital Essen, Department of Addictive Behaviour and Addiction Medicine, Medical Faculty, University of Duisburg-Essen, Virchowstrasse 174, 45147 Essen, Germany; henrike.schecke@uni-due.de (H.S.); Michael.Specka@lvr.de (M.S.); norbert.scherbaum@uni-due.de (N.S.); 3 Faculty of Social Sciences, Department of Health Sciences, Tampere University, 33014 Tampere, Finland; irja.nieminen@tuni.fi (I.N.); eija.paavilainen@tuni.fi (E.P.); 4 Etelä-Pohjanmaa Hospital District, 60220 Seinäjoki, Finland; 5 Department of Psychology, The Maria Grzegorzewska University, Szczęśliwicka 40, 02-353 Warsaw, Poland; lzablocka@aps.edu.pl; 6 Department of Psychology, University of Warsaw, Stawki 5/7, 00-183 Warsaw, Poland; malgorzata.wozniak-prus@psych.uw.edu.pl; 7 Faculty of Health and Life Sciences, Oxford Brookes University, Jack Straw’s Lane, Oxford OX3 0FL, UK; jvappleton@brookes.ac.uk (J.V.A.); sbekaert@brookes.ac.uk (S.B.); 8 School of Medicine University of St Andrews, N Haugh, St Andrews KY16 9TF, UK; gz37@st-andrews.ac.uk (G.Z.); amb30@st-andrews.ac.uk (A.B.); 9 Mental Health and Social Sciences Research Laboratory, Groupement Hospitalier Universitaire, Psychiatrie & Neurosciences (GHU-PARIS), 258 Rue Marcadet, Bât N, 2ème étage, 75018 Paris, France; Emmanuelle.JOUET@ghu-paris.fr; 10 Synergia s.r.l., Via Settala 8, 20124 Milan, Italy; gvigano@synergia-net.it; 11 Division of Psychiatry, University College London, 149 Tottenham Court Road, London W1T 7NF, UK

**Keywords:** digital health, e-learning, professionals, child maltreatment, multidisciplinary

## Abstract

The responsiveness of professionals working with children and families is of key importance for child maltreatment early identification. However, this might be undermined when multifaceted circumstances, such as the COVID-19 pandemic, reduce interdisciplinary educational activities. Thanks to technological developments, digital platforms seem promising in dealing with new challenges for professionals’ training. We examined a digital approach to child maltreatment training through the ERICA project experience (Stopping Child Maltreatment through Pan-European Multiprofessional Training Programme). ERICA has been piloted during the pandemic in seven European centers involving interconnected sectors of professionals working with children and families. The training consisted of interactive modules embedded in a digital learning framework. Different aspects (technology, interaction, and organization) were evaluated and trainers’ feedback on digital features was sought. Technical issues were the main barrier, however, these did not significantly disrupt the training. The trainers perceived reduced interaction between participants, although distinct factors were uncovered as potential favorable mediators. Based on participants’ subjective experiences and perspectives, digital learning frameworks for professionals working with children and families (such as the ERICA model nested in its indispensable adaptation to an e-learning mode) can represent a novel interactive approach to empower trainers and trainees to tackle child maltreatment during critical times such as a pandemic, and as an alternative to more traditional learning frameworks.

## 1. Introduction

The COVID-19 pandemic, declared in early 2020, committed governments worldwide to endorse containment measures and restrictions such as quarantine, lockdown, and social distancing with the suspension of all public events and some commercial activities [1]. These measures had a significant social and psychological impact on vulnerable populations, such as those at risk of child abuse and neglect [2,3,4,5,6], as well as on healthcare workers [7]. Since professionals working with children and families, such as health care workers, teachers, and social workers, have a key role in the early identification of child maltreatment [8], this may have had an impact on the responsiveness of professionals in the reporting process of at-risk situations. In addition, in most countries worldwide, the COVID-19 containment measures involved the shutdown of educational institutions, including schools, kindergartens, universities, and of non-essential primary and community health and social care facilities. As a result, face-to-face educational activities, including professionals’ training, became unfeasible. Thus, the COVID-19 pandemic presented a new challenge for professionals learning programs within the medical, social, and psychological areas of education, prioritizing the need to develop, evaluate, and disseminate e-learning interventions for people working with children and families [9]. For instance, the World Health Organization (WHO) had already developed the WHO Academy, a lifelong learning center about global health, which through the means of the latest technologies provides in-person and blended learning programs for health professionals worldwide [10].

A Cochrane Review by Vaona and colleagues [11] showed that e-learning might be equally successful in improving healthcare professionals’ knowledge, skills, and behaviors compared to traditional learning. Nonetheless, these outcomes cannot be taken for granted and the need for developing good practices in distance learning is crucial [11]. Available evidence emphasizes the importance of investing in accurate conceptualization and evaluation of e-learning features in order to develop high-quality e-learning training for professionals [12]. Considering various levels of interventions required by child maltreatment identification, management, and prevention, what became clear with technological advancements, especially in the era of COVID-19, was the need for a novel framework based on knowledge, interactions, and perceptions of professionals of interconnected sectors mediated through proper and effective use of digital tools [13]. A multidisciplinary and interprofessional approach is at the heart of an efficient child abuse prevention program, where communication and collaboration between professionals are vital to the early and rapid identification of at-risk situations. This poses a particular challenge in the development of e-learning training [14]. Thus, to enable the empowerment of professionals working with children and families in an adaptable and responsive supportive system, there is a need to collect baseline data on pre-training knowledge, pilot the training, and assess outcome measures together with the evaluation of the digital interaction embedded in the training [15].

Over the years, some programs, such as the PROCHILD–*PROtection and support of abused CHILDren through multidisciplinary intervention* [16] have been developed and piloted across Europe in the attempt to provide tools for professionals to recognize and prevent child maltreatment. With the development of technology, the audience of these programs has broadened and some examples of digital training about the reporting process of child maltreatment for health care practitioners [17], child-care professionals [18], and teachers [19] have originated.

Focusing on the specific perspectives of both training participants (trainees) and organizers (trainers), we have purposely designed and piloted during the COVID-19 pandemic an innovative e-learning training for professionals working with children and families, involving seven partners from different European countries—England, Finland, France, Germany, Italy, Poland, and Scotland. The training is part of the pan-European research project—ERICA (*Stopping Child Maltreatment through Pan-European Multiprofessional Training Programme: Early Child Protection Work with Families at Risk*), funded by the European Commission Rights, Equality and Citizenship programme. The ERICA project aimed to prevent and tackle child maltreatment through the improvement of the expertise of professionals, by building training that also provided an opportunity for multidisciplinary professionals to converge their knowledge of child maltreatment and their skills to identify minors living in families with at-risk conditions [20]. Therefore, the purpose of this paper is to describe the experience of the ERICA project on child maltreatment with a particular focus on its digital approach. Due to the unusual circumstances of the pandemic, an e-learning version of the ERICA learning program on child maltreatment was necessary. We hypothesized that, under these specific circumstances, this adaptation may represent a key element in the process of delivering and also promoting the novelty value of the ERICA training. Therefore, trainers’ subjective perspectives on specific advantages and disadvantages of the ERICA digital approach on child maltreatment prevention training were explored. This is crucial, especially given the persistence of the pandemic, to promote relevant good practices across Europe.

## 2. Materials and Methods

### 2.1. Study Design

The ERICA Consortium designed and developed training on child maltreatment for professionals. The training was developed in an iterative process made up of seven interactive modules plus an evaluation module.

To pilot the training, 15 professionals were involved as appointed trainers across different countries. In detail, three trainers each from Finland and France, two from England, Germany, Italy, and Scotland, and one from Poland, carried out the ERICA digital training, combining specific expertise in the child maltreatment field and experience as a training leader. The trainers productively provided the consortium with feedback on the course of the training. Most of the ERICA trainers were women (80%). Their background was heterogeneous, including education facilitators, professionals from health sciences and nursing education fields, pediatric nurses, and social workers employed in child protection services in collaboration with psychologists and psychiatrists. In addition, two out of seven partners also involved a trained user-trainer, i.e., a person who had a history of child maltreatment and who could share her/his personal experiences with participants.

In each center, the trainers dealt with two cohorts of participants working with children and families who were administered the different training modules in order to generate the final version of the ERICA program. Across all seven countries, the two cohorts involved 328 individuals who participated in the pilot study. Both cohorts were requested to fill in a pre-measurement online survey of child maltreatment knowledge and skills, before going through the program.

The training was followed by the collection of structured feedback from trainers and trainees. Thus, two post-measurements (at the conclusion of the training and at one-month follow-up) via online survey were recorded regarding knowledge, skills, and satisfaction and including recommendations within the scope of child abuse and neglect management and prevention for potential changes to the training. The feedback from both cohorts was then incorporated and the modules modified accordingly. Further details on the development of the training and preliminary quantitative and qualitative findings are available elsewhere [20].

### 2.2. ERICA Learning Framework Content

The content of the ERICA interactive training was based on “Intended Learning Outcomes” that were defined for each module by selecting knowledge and skills that were considered to be essential needs for professionals dealing with child maltreatment prevention and management. These were previously evaluated by an online survey among professionals working with children and families. The identified training key topics included child development, early signs of maltreatment, risk and protective factors, risk assessment tools, and potential interventions to tackle child maltreatment. These are summarized in Table 1 (see Appendix A for a more in-depth description). Related content, along with its graphic design, was handled according to up-to-date scientific evidence in each topic area and the practical relevance for the trainees. 

Some cross topics, such as responses to the COVID-19 pandemic consequences, were also incorporated throughout the modules [20]. 

Originally, the content was created in English. Then, it was translated into all languages of the ERICA Consortium member states and adapted to national requirements and cultural characteristics of the different countries.

### 2.3. Adaptation to an E-Learning Mode and Qualitative Framework

Although originally planned to be delivered face-to-face, the ERICA consortium considered that a digital version was needed and inevitable during the ongoing pandemic. Thus, the ERICA training was embedded in an e-learning framework to comply with current needs based on regulations about contact restrictions. Moreover, given the international, multicentric, interdisciplinary, and multicultural approach of the ERICA project across Europe, the digital approach seemed particularly suited to facilitate coordination and communication between centers, as well as the sharing of materials and feedback and the implementation of the training in a rather synchronous way among different countries. A blended learning approach was followed, with synchronous training sessions in video conferences held by appointed trainers and asynchronous provision of materials for review and more in-depth study. In addition, a guideline for the trainers and a set of presentation slides were created (further details are available in Supplementary Materials) [21].

We carefully reviewed the digital delivery of the training according to the existing circumstances demands in order to supply an accurate evaluation of the ERICA program. Specifically, we evaluated the different digital features of the training on child maltreatment since these may influence personal subjective perspective and the potential of this innovative e-learning program aimed to improve professionals’ knowledge and skills.

Therefore, a survey on digital features of the ERICA training was specifically designed and administered to the appointed trainers at each center before the end of the project. This was made up of 30 questions, developed from the literature on digital training implementation for professionals with a particular focus on its domains [22]. Ethical approval details are reported elsewhere [20].

The first part of the digital evaluation asked for details about the country where the ERICA training was carried out, while the second part comprised yes or no, open-ended, and multiple-choice answers in response to a series of statements or questions on digital features. These encompassed reasoning across three different but interrelated sections in an attempt to reveal experiences that can be difficult to quantify: Technology, Interaction, and Organization. First, Technology was defined through a set of questions focusing on technical issues. These were aimed to draw attention to potential barriers and contextual factors that might disrupt the training (e.g., connection-related).

Then, the evaluation considered Interaction, as the assessment of the exchange and relationship between trainers and trainees through the digital platform. Different inputs were provided in order to gather trainers’ subjective perspectives on the quantity and quality of the interactions both within the trainees’ group and between the group and the trainer.

Finally, Organization dealt with how the child maltreatment training’s structure was used, both in terms of timing and institutional-related issues.

Across the three sections, statements and questions first dealt with general understanding, and afterwards with more specific issues. In addition, space for free text comments based on participants’ knowledge, impressions, and additional perceptions was included. Respondents were free to skip any question they considered unsuitable. The questionnaire was piloted with a small group of medical students to ensure that the questions were clear and comprehensive. The full list of questions, specifically referring to technology, interaction, and organization inter-related domains, is available in Appendix B.

The survey was then distributed to ERICA trainers in all seven countries using Google Modules on 20 May 2021, and all answers were collected by 20 June 2021.

Survey responses from center-specific representatives were analyzed using content analysis, by searching for shared patterns and themes among different centers to deepen the understanding of the trainers’ experience. A coding framework, with different codes grouped into categories according to their reciprocal relationship in terms of content and context, was generated in order to label apparent themes according to standard guidelines. Based on the available amount of text, following a two-way strategy, combining content analysis top-down and a grounded bottom-up approach, we double-checked that categories were reflective of the purpose of the research, exhaustive, mutually exclusive, sensitizing, and conceptually congruent.

Recurring issues were clustered reaching a consensus among team members for the final narrative. In particular, practical recommendations for the specific digital learning framework were derived according to domains’ facilitators and obstacles specifically distinguishing between positive and negative factors. This facilitated to split patterns related to particular contexts from those shared across the various cultural environments. In addition, a selection of excerpts from the respondents was extracted in order to depict shared patterns or to highlight potential differences.

## 3. Results

Despite the restrictions due to the COVID-19 pandemic, the ERICA project expected time frame (2019–2021) was warranted by the e-learning mode of the model, which allowed the synchronous implementation of the training and the collection of data in seven European countries. However, additional data on subjective experiences were gathered concerning the specific digital features of the training. Trainers from all seven ERICA centers (n = 15) provided their feedback in the digital evaluation survey. Therefore, different professional subjective perspectives complemented each other based on their heterogeneous background, as workers in inter-connected professional sectors (i.e., education, health sciences, mental health, and social care). A top-down strategy from domains proposed, combined with a grounded bottom-up approach, generating newly identified themes from transcripts, produced a consistent picture, though based on a limited amount of textual material.

Table 2 shows categories and subcategories that emerged from the content analysis in terms of technology (internet connection; technical abilities; trainer’s role), interaction (contextual factors; individual characteristics; group issues), and organization (timing; digital settings; pandemic issues).

Internet connection issues were among the main barriers to the digital form of the training, about 40% of trainers considered this issue as the main problem with the digital platform (category 1.1 Internet connection):

*“People disconnected and reconnected repeatedly during the training session.”*

Indeed, major difficulties were encountered when videos were streamed or when breakout rooms were used. Further infrastructure issues were also reported due to occasional interruptions in terms of audio problems, videos broadcasting, and institutional network disruptions.

On the other hand, trainers had more difficulties in the first training session rather than in the subsequent ones, since most of them were not entirely confident with the technology used. In particular, some trainers reported technical problems as related to participants’ expertise with digital tools (categories 1.2 Technical abilities and 1.3 Trainer’s role/1.3.2 Grouping issues):

“*The problem of dividing people into groups was connected with the low technical abilities of the participants. Some of them didn’t deal with the selected digital platform earlier and had many difficulties.*”

“*We did have a problem with the videos, but trainees watched them individually on their PCs. The fact that trainees couldn’t join the breakout rooms due to the lack of experience or using Teams, or them joining us via telephone or iPad, was addressed by allowing those trainees to stay in the main meeting room and calling in a new breakout room.*”

Overall, according to respondents, technical difficulties did not significantly disrupt the training. However, it is worth mentioning that 28% of trainers admitted having to cut small parts of the training because of technical issues.

Furthermore, according to their experience, the trainers perceived during the digital training a reduced interaction both between themselves and trainees and within the group as compared to similar training in non-online settings. This was specifically mentioned in relation to informal interactions (category 2.1 Contextual factors/2.1.3 Atmosphere):

“*Interaction among participants and between participants and trainers were limited, especially more “informal” interactions in breaks etc., which usually lead to a more familiar work atmosphere during face-to-face trainings.”*

As a result, trainers had to drive the discussion multiple times. Trainers’ competence and skills represented key elements for the digital training (category 2.2 Individual characteristics/2.2.3 Trainers’ skills in engaging participants):

“*The participants had many questions and a great will to share their experience to discuss and also to talk to the trainer about their experience. The trainer is a very experienced and open specialist so I think it was an important factor that participants were so active. There was more activity between trainer and trainees.”*

The effect attributable to the lack of a warm atmosphere was also mitigated since some trainers reported they perceived high-quality human contact within the trainers’ group (categories 2.1 Contextual factors/2.1.1 Location/environment; 2.2 Individual characteristics/2.2.2 Trainers’ team skills):

“*Because of the quality of the content and the fact that trainers were together in the same room and not at home made the training session successful.*”

In addition, an enhanced interaction was perceived by the trainers when the ERICA training benefited from the use of additional digital components of the platform (e.g., chat function) in order to encourage friendly communication among participants (category 2.1 Contextual factors/2.1.2 Digital tools):

“*Having chat box was incredibly beneficial during the training, as each trainee was able to raise points that were then discussed by the trainers. This increased trainer and trainee interaction.*”

Similarly, the interaction was depicted by comments by trainers on their ability to lead small group activities dealing with sensitive topics related to child maltreatment prevention and management of at-risk scenarios (category 2.3 Group issues/2.3.3 Privacy):

“*Small group activities allowed a level of privacy that being in the same room would have not allowed. This was particularly important when some of the trainees decided to reveal their own personal stories of maltreatment that they did not feel comfortable mentioning the whole group.”*

About 70% of trainers described their cohorts made up of people who did not know each other which may have affected interaction, especially considering the sensitive and emotional matters related to child abuse discussed. Interestingly, as with technical problems, the second training sessions proved less problematic also from the interaction point of view, at least according to about 40% of trainers.

Finally, the organization section of the survey provided details on timing as well as on the digital infrastructure and the choice of platform. Many advantages were identified in terms of the training schedule and the opportunity it provided for remote participation. However, feedback suggested that the e-learning adaptation had a significant impact on the number and quality of breaks for some participants, consistently with a lack of interaction between participants during breaks in comparison to face-to-face learning frameworks (category 3.1 Timing):

“*Different advantages were related to the use of a digital platform (trainees in remote geographical locations, or trainees who had to be at work during the training session), which increased the attendance. However, there was a lack of human communication and relaxation during the breaks because it was done online.*”

As regards the digital infrastructure, three different platforms for training were selected according to the familiarity of both trainers and trainees and available institutional resources in each country: Cisco Webex^®^ (*n* = 1), Microsoft Teams^©^ (*n* = 4), and Zoom^©^ (*n* = 2), supplemented by Moodle^™^. About 70% of trainers declared they chose the platform based on institutional agreements with a particular provider. None of the trainers decided to change platform between the first and the second session of training although 29% of respondents described themselves as “not satisfied” with the use of the digital platform. In particular, a platform change was not always feasible, possibly due to institutional requirements (category 3.2 Digital platform):

“*No possibility to change. In the fields where the professionals stemmed from, Zoom is more used than Teams or other digital platforms*”.

However, from a technical point of view, the different platforms appeared comparable. In general, when answering questions on organization, trainers remarked on the reluctance of trainees to spend more time online, probably because of the spread of smart working since the beginning of the COVID-19 pandemic (category 3.3 Pandemic issues):

“*Because of the pandemic situation and the home office, people were tired with online working. They told us they needed personal contact and would be more happy with that form of the training*.”

Finally, we uncovered slight differences in country reports that might derive from the different cultural specificities. For example, Finland, Poland, and England respondents felt that the digital platform affected primarily the interaction among trainees and less the interaction between trainers and trainees. Similarly, but from a different perspective, trainers from Italy, Scotland, and France reported they had more interaction with trainees as compared to non-digital settings, since they felt more responsible in keeping interaction lively.

## 4. Discussion

### 4.1. Strengths and Weaknesses of the ERICA Digital Training

The ERICA project model offers feedback on digital aspects of the evidence-based professionals’ training for child maltreatment prevention that are possibly useful to develop and share e-learning good practices at the European level even for neighboring fields in the ongoing pandemic situation. Comments and points of view of both ERICA trainers and trainees provide useful guidance to all those who are in the process of building digital training within a novel learning framework. The adaptation of ERICA to the e-learning mode is likely to have a key role in supporting and promoting the value of the learning model, in particular under specific circumstances which make a digital version of the training essential. Therefore, based on the ERICA project experience, we identified several advantages of e-learning as compared to traditional face-to-face learning in providing professionals with an innovative learning framework.

First, the e-learning mode facilitated the implementation of a European, multicentric, interdisciplinary and multicultural program, allowing easier communication between centers, the synchronous feasibility of the training, the delivery of comments and feedback, the immediate availability of additional material from the shared platform, and the quick exchange of common issues and tentative solutions.

This seems crucial according to slight differences we found in the subjective experiences of the professionals across the seven European countries. Indeed, professionals working with children and families in different national contexts are likely to face varying local situations, grounding on different cultural specificities, thus influencing their behavior and interaction skills. According to available evidence, this may, at least partly, relate to cultural competency and cultural burden in the process professionals involved are in charge of, including a simultaneous assessment of the complex influences of child development, culture and child-rearing practices and the risk of harm to the child [23]. This should be taken into account when designing a novel learning framework for child maltreatment, especially considering the adaptation to an e-learning mode, since interaction skills are likely to play a key role in the impact of the training, by generating a positive perception of the program.

Second, considering the COVID-19 pandemic restrictions, the digital design of the ERICA training gives professionals the opportunity to test their skills and responsiveness in the child maltreatment evaluation and reporting process. In addition, this may facilitate the identification of strategies that minimize the risk of potentially harmful consequences of practices such as mandatory reporting when these are done outside a structured framework [10]. Nonetheless, the specific circumstances may have reduced the potential impact of the digital learning framework, as shown by themes identified from the content analysis. This seems mainly linked to a lack of personal contact that study participants reported as related to the sudden increase and persistence of online working.

On the other hand, engaging in e-learning tasks increases both trainees’ and trainers’ practical teaching skills, especially technical abilities, thus empowering them in the use of different digital tools. Professionals’ digital empowerment for child protection is likely to emphasize the importance of building knowledge, attitudes, skills, and responses to contrast child maltreatment. This may contribute to the effectiveness of this novel digital framework for training. Moreover, adopting new tools may give both trainers and trainees a positive experience overall and break down their preconceptions about digital training. Specifically, participants reported that they benefited from a sense of privacy that this digital training, especially as embedded in small group activities, is able to provide them with, even when considering personal, sensitive topics which may have been prompted by the training program. Being at home and not in the same physical place may generate closeness and intimacy among participants. In particular, issues related to violence and child maltreatment usually arouse feelings that participants have to cope with and managing different emotions may appear easier and safer during online training.

In addition, trainers’ feedback revealed that especially from the trainees’ point of view, digital training gives additional freedom in terms of choosing a place, time, and pace for learning through both synchronous activities and extra material made available by trainers [22,24]. This easier access is particularly well-suited considering the inclusion of professionals from different institutions and settings across a wide range of specialties with different work schedules, even offering an important opportunity to contribute to contrasting child maltreatment throughout the COVID-19 pandemic. Indeed, while trainees may benefit from additional resources to deepen their learning, the program acquires reliability, flexibility, and accessibility for learning. Participants from different parts of each country, including urban, rural, and remote areas, could take part in the training without traveling. This seems promising independently of the pandemic situation. Moreover, the costs of using a digital platform are significantly lower compared to face-to-face learning, enabling to teach even smaller groups [22]. Globally, this kind of change of attitudes, within a novel e-learning training framework, appears crucial in terms of successful development and implementation of high-quality digital learning in child maltreatment.

However, according to themes identified from participants’ feedback, some technical challenges should be acknowledged. Technical issues that were reported by participants seem attributable to a lack of experience with digital platforms. These problems are probably attributable to the suddenness of the “digitalization” of training in the COVID-19 pandemic, which has led many to unexpectedly use digital platforms they had very little, if any, experience with. In the foreseeable future, these technical flaws should at least in part diminish. Indeed, in the second cohort trainers clearly reported they had become more familiar with digital tools and training sessions, which were generally carried out more smoothly. Indeed, also the available evidence confirms that technical problems can disappear completely once trainers develop experience in online teaching [25]. Thus, the promotion of good training practices for child protection should consider trainers’ familiarity and compliance with digital platforms, supporting the development of trainers’ technical skills alongside self-learning [26].

Furthermore, some problems with the technical infrastructure were sometimes detected in line with previous evidence [22,27], thus requiring a more structured approach and the need to involve the institution in the unavoidably cross-disciplinary child protection training process. Technical aspects should be considered in light of the opportunity of enhanced communication and collaboration between trainees meeting in a digital environment. In particular, the ERICA training was designed as an interprofessional e-learning training, since child protection requires an exchange between different healthcare, social care, and educational professionals of interconnected sectors. Professionals’ exchange seems critical to pursue the establishment of a proactive network to have an impact on various spheres of the caregiver-child duo together with community-level risk factors. These may include the absence of daycare, school, and community-based programming for children and adolescents, a central problem during the COVID-19 pandemic, as well as the loss of caregiving supports and socio-economic stress, burnout, and mental health challenges, a possible consequence of the caregiver’s chronic and overwhelming parenting stress [9]. Interprofessional training settings mirror work environments, where contact and cooperation are essential features of a successful trainers-trainees interplay as well as within the trainers’ group. These characteristics are particularly challenging when training on child maltreatment has to be developed as e-learning since a perceived reduction in interaction in the group was pointed out as an important criticism as elicited from the content analysis of trainers’ subjective experiences. However, this digital learning framework embraces a supportive learning context, where reflective supervision and the use of experiential or simulation-based training may be able to elicit emotions within a system that is likely to enhance the development of emotional intelligence competencies [28]. Indeed, though based on a small number of sessions, the interprofessional nature of the ERICA e-learning training endorses openness with colleagues and an organizational culture that values acceptance, thus promoting the relatedness and the emotion regulation of professionals [29]. Therefore, considering that the emotional aspect in research and practice could be quite complex and demanding [30], high-quality e-learning training should deal with strategies to support interaction and social learning between trainees, along with the role of trainers in providing appropriate directions [31].

For this purpose, traditional activating teaching methods can also be used in digital settings [32]. The training design should support trainers in their role as panelists to navigate trainees’ interactions in some child maltreatment scenarios, including interactive virtual “flipcharts”, short surveys, or embedded case study simulations. These may contribute to promoting interaction and enlivening the training [31]. In addition, based on the perception of social presence, especially considering the COVID-19 pandemic’s consequences on how we relate to each other and emotional contagion [33], despite technical difficulties small groups work appeared a valid tool to build a friendly environment for the e-learning model of training on child maltreatment and sensitive social issues. This opportunity is offered by most digital platforms, but a specific technical infrastructure is needed. Moreover, our qualitative findings suggest that the use of the chat function for exchanges, questions, comments, and remarks can prove useful, since direct messaging between trainees can also create more informal communication that occurs, for example, during breaks at face-to-face training sessions.

### 4.2. Recommendations from the ERICA Novel E-Learning Framework

In order to avoid difficulties experienced during the training, ERICA trainers’ feedback and identified themes from content analysis based on their subjective experience were used to develop a series of recommendations. First, within a novel e-learning for professionals’ training, framed within an interdisciplinary field such as child maltreatment management and prevention, the utilization of a well-known digital platform seemed to be advantageous since trainers and trainees were both familiar with how it worked, particularly in the COVID-19 era when most people were working remotely. Furthermore, accomplishing a short rehearsal of the lesson/lecture on the digital platform avoided many potential technical problems before the training (media streaming, time limitations, etc.). In addition, in order to build mutual interactions, components such as breakout rooms, which allow participants to work in smaller groups, proved effective with an enhanced sense of privacy, whereas more communal settings make it easier for trainees to lose focus and attention. This made professionals more prone to exchange individual perspectives even considering personal, sensitive experiences as regards distinct but inter-related themes, including child maltreatment signs and consequences, a better understanding of risk factors and their assessment, as well as a wider perspective into potential evidence-based intervention strategies for child protection. Moreover, another useful option can be a chat box used as a questions and answers section which may provide the trainers with the opportunity to encourage participants to ask written questions during the learning session. This works well in making the session more interactive as well as in ensuring that participants are actively following the training and putting their competencies at stake to enhance related skills without feeling overwhelmed by emotional issues intrinsic to child maltreatment topics. Using external software such as search engines’ and trademarks’ shared modules and collaborative whiteboards for brainstorming can be effective to make the training more diverse benefiting from trainers and trainees collaborative thinking [34]. In addition, some digital platforms enable trainers and trainees to exchange materials (e.g., documents, media, meeting notes) even after the training, thus providing a designated space (e.g., team’s space).

In general, due to characteristics such as familiarity, understanding, knowledge, and experience with a specific digital platform, designing the e-learning training according to that digital platform assures increased interaction and fewer unexpected technical problems. Available evidence on the association between educational benefits and e-learning mode for novel learning frameworks, as compared with traditional educational programs, are mixed, ranging from small positive effects to no substantial effect [11]. Trainees’ seniority might partly explain this discrepancy since e-learning tools fare better in younger populations. 

However, despite a considerable amount of research in the digital healthcare field dealing with students’ education as well as with health preventive strategies, high-quality evidence on e-learning for professionals remains scarce [22,35,36,37]. Therefore, further research is needed especially considering training for professionals—and more specifically for professionals working with children. It is essential thus to explore their compliance with digital systems, their synergy with trainers, and interaction with other professionals during training sessions using robust and well-resourced data flows. The ongoing COVID-19 pandemic situation indeed poses an additional burden [7].

### 4.3. Limitations

We should acknowledge some limitations. Although the pandemic situation encouraged the design of a more targeted approach embedding the ERICA learning program in a digital framework, the complex working situation might have had an impact over the course of the study. This is likely to have involved recruitment and sampling strategies making participation in the training program more complex. In addition, a potential sampling bias might have occurred in terms of participant characteristics (e.g., gender). Similarly, recruitment procedures may have had an effect on trainers’ subjective perspectives influencing the burden related to bringing together the trainees in different sessions and keeping the interaction lively, possibly resulting in impaired motivation and behavior.

In addition, focusing on the structure of the digital evaluation survey, dichotomous situations which trainers were provided with might have offered them less opportunities to disclose additional information about their subjective experiences. However, this was somehow counterbalanced by a set of open-ended questions, in which participants were provided with the opportunity to list and comment on further individual perspectives. As a whole, data gathered were possibly not rich enough to produce deep and more detailed thematic analyses, though meaningful results were grounded in derived categories and representative excerpts from respondents.

## 5. Conclusions

Although our study design does not allow us to draw any firm conclusions on the superiority of an e-learning approach, this seems suitable for both traditional and novel learning frameworks thanks to the interactive properties of advanced technology, even during pandemic times. Based on the ERICA pan-European experience on child maltreatment training for professionals, the designed digital framework for learning can effectively contribute to developing new strategies to support multi-layered settings where a multidisciplinary and interprofessional approach is needed. Finally, this promising approach may represent an engaging option that emphasizes the strengths of e-learning under certain circumstances, i.e., a valid alternative when more traditional learning is limited by a range of different measures that prevent to carry out face-to-face educational activities.

## Figures and Tables

**Table 1:** Child protection-related key topics of the ERICA digital training interactive modules.

ERICA Key Topic	Description
Child development	Focus on neurodevelopmental and psychopathological consequences of abuse for children and adolescents.
Early signs of maltreatment	Focus on knowledge about early signs of sexual abuse as well as emotional abuse and neglect, including prenatal neglect.
Risk and protective factors	Individual and family characteristics that may influence child safeguarding concerns, also involving considerations about disadvantaged communities and cultural/ethnic differences in terms of family life, coping strategies and parenting practices.
Risk assessment tools	Encompassing assessment techniques used where there are potential safeguarding concerns.
Potential interventions to tackle child maltreatment	Professionals’ intervention skills in different settings, e.g., when talking to an abusive parent; when supporting the whole family; when approaching the child of a specific age group.

**Table 2:** Main categories from content analysis of ERICA digital trainers’ responses.

Domain	Categories	Subcategories
**1. Technology**	1.1 Internet connection	1.1.1 Stability
		1.1.2 Slowness
	1.2 Technical abilities	1.2.1 Individual expertise
		1.2.2 Data stream sharing
	1.3 Trainer’s role	1.3.1 Type of individual account
		1.3.2 Grouping issues
**2. Interaction**	2.1 Contextual factors	2.1.1 Location/environment
		2.1.2 Digital tools
		2.1.3 Atmosphere (formal/informal)
		2.1.4 Training advertisement
	2.2 Individual characteristics	2.2.1 Participant characteristics & profession
		2.2.2 Trainers’ team skills
		2.2.3 Trainers’ skills in engaging participants
		2.2.4 Trainers’ skills in technical expertise (participants’ needs in terms of technical assistance)
	2.3 Group issues	2.3.1 Size
		2.3.2 Contents recollection
		2.3.3 Privacy
		2.3.4 Timing
**3. Organization**	3.1 Timing	3.1.1 Schedule and breaks
		3.1.2 Duration
	3.2 Digital settings	3.2.1 Type of platform
		3.2.2 Institutional mandatory requirements
		3.2.3 Trainers synergy
	3.3 Pandemic issues	3.3.1 Current circumstances and restrictions
		3.3.2 Online working
		3.3.3 Personal contact and communication

## Data Availability

Data are available upon request.

## Supplementary Materials

Training materials are available on the ERICA website (https://projects.tuni.fi/erica/). Accessed on the 20 November 2021.

## Appendix A

**Table A1:** Detailed ERICA training topics.

1. Introduction	Description of the main purpose of the training. Understanding the structure of the course, the modules, and how they can work through the material. Understanding how to give feedback, discuss points, clarify something that is not clear.
2. Child development and consequences of maltreatment	Overview of theories of prenatal, infant and child development (intellectual, emotional, physical, psychological). How different types of maltreatment can influence development. More critical and sensitive periods to maltreatment and other heterogeneities to be taken into account.
3. Early signs of maltreatment and neglect	Common recognizable signs of maltreatment in children at different developmental stages. Typical signs of certain types of abuse. Distinguish between normal phases of development and what may reveal a problem. Understanding that various forms of maltreatment may intersect, and signs may be complex to identify.
4. Risk factors	Family and parent/guardian risk factors (e.g., intergenerational issues, mental health, substance misuse). How risk factors can manifest in parenting practices. Differences/heterogeneity in risk factors across contexts, including cultural differences in parenting practices.
5. Risk assessment tools	Feel confident in using some common risk assessment tools for different kinds of child maltreatment. Difficulties of generalising checklists across contexts. Be aware of the strengths and weaknesses of risk assessment tools and use them with a critical perspective.
6. Intervention techniques and skills	Be able to judge the appropriateness of interventions with the children and their families and know how to interact with other agencies. Become confident to use some common intervention techniques that are suitable for different situations. Learn techniques to effectively engage with families. Be able to provide support at key vulnerability points in children’s lives.
7. Protective factors	Be aware of various protective factors for different kinds of child maltreatment covering family and parenting characteristics, socio-demographic factors, ethnicity and cultural factors, large social networks, and wider protective structures (e.g., inter-agency working, child engaging in different non home contexts). Reflect on whether these protective characteristics are amenable to intervention.
8. Syndemic responses to global pandemic	Have knowledge about how pandemic responses can exacerbate existing risk in families. Feel confident about spotting and assessing possible maltreatment in the era of physical distancing and how inter-agency working evolves. How engagement with children and families can be adapted, maintained, and evolve through new means such as technology development.
9. Evaluation	How the learner used the material. What was useful and what was less useful. What they missed and needed more of. Impressions on mode of delivery (e.g., slides, videos, live workshops etc.). Whether they felt if it was well adapted to their local context.

## Appendix B

**Table A2:** Feedback on digital training in the ERICA Project.

**Question**	**Type of Answer (Open-Ended, Dichotomous, Multiple Choice)**	**Responding Centers (No.)**
**Part 1/3**—Technical problems		
1. During the first ERICA training did you have any technical problems with the digital platform?	Dichotomous	7
2. Did the same problems arise in the second ERICA training?	Dichotomous	7
3. If you answered no, were they primarily: Audio Problems, Image problems, Network Disconnection, or Trouble broadcasting videos?	Multiple choice	5
4. Did this problem interrupt significantly the session?	Dichotomous	6
5. Were you forced to change or cancel parts of the lesson because of technical problems? (e.g., not show a video, not do a group activity…)	Dichotomous	7
6. Please list and comment any other technical problems that come to mind.	Open-ended	5
**Part 2/3**—Interaction		
1. Which of the following sentences better describes interaction during your training? (a) There was more interaction between trainer and trainees (b) There was more interaction among trainees (c) There was less interaction between trainer and trainees (c) There was less interaction among trainees	Multiple choice	7
2. Please add a brief comment on interaction in the training	Open-ended	7
3. Were you able to do small-working group activities?	Dichotomous	7
4. Do you think small-group activities worked well despite the digital setting?	Dichotomous	7
5. Why do you think small group activities worked well?	Open-ended	5
6. Was the trainer asked questions on the contents of the training?	Dichotomous	7
7. Did the trainees know each other?	Dichotomous	7
8. Were the trainees co-workers?	Dichotomous	7
9. Please briefly describe the background of the trainees (how were they recruited, what was the connection between the partner and the trainees…)	Open-ended	7
10. Did you feel like the participants were more active (cognitively more present, more attentive, felt more responsible…) in the training compared to how trainings not on digital platforms usually are, in your experience?	Dichotomous	7
11. In which training session was interaction better?	Multiple choice	6
12. In which module do you feel like there was more interaction?	Multiple choice	7
13. How was the chat used?	Open-ended	5
14. Please list and comment any other problems with interaction that come to mind.	Open-ended	7
**Part 3/3**—Organization		
1. Did the digital platform influence the duration and the temporal organization of the training?	Dichotomous	7
2. What platform did you use?	Multiple choice	7
3. Did your center or university suggest the platform?	Dichotomous	7
4. Were you sufficiently satisfied with the platform?	Dichotomous	7
5. Did you change digital platform between cohort 1 and cohort 2?	Dichotomous	5
6. Was the change due to technical or organizational problems?	Dichotomous	3
7. Did you provide trainees with any organized asynchronous activity (e.g., videos to watch home, extra material to look at before or after the training)?	Dichotomous	7
8. Please list and comment any other problems with organization that come to mind.	Open-ended	3
9. General comments on Training on a Digital Platform	Open-ended	4
